# Cellulose Acetate Phthalate and Antiretroviral Nanoparticle Fabrications for HIV Pre-Exposure Prophylaxis

**DOI:** 10.3390/polym9090423

**Published:** 2017-09-07

**Authors:** Subhra Mandal, Karl Khandalavala, Rachel Pham, Patrick Bruck, Marisa Varghese, Andrew Kochvar, Ashley Monaco, Pavan Kumar Prathipati, Christopher Destache, Annemarie Shibata

**Affiliations:** 1 School of Pharmacy and Health Professions, Creighton University, 2500 California Plaza, Omaha, NE 68178, USA; SubhraMandal@creighton.edu (S.M.); PavanKumarPrathipati@creighton.edu (P.K.P.); ChrisDestache@creighton.edu (C.D.); 2 Department of Biology, Creighton University, 2500 California Plaza, Omaha, NE 68178, USA; karlkhandalavala@creighton.edu (K.K.); Rachelpham@creighton.edu (R.P.); mvarghese@creighton.edu (M.V.); andrewkochvar@creighton.edu (A.K.); ashleymonaco@creighton.edu (A.M.); 3 Dana-Farber Cancer Institute, Harvard University, Boston, MA 02215, USA; PatrickT_Bruck@dfci.harvard.edu

**Keywords:** cellulose acetate phthalate, anti-retroviral, nanoparticles, thermosensitive gel, HIV, pre-exposure prophylaxis

## Abstract

To adequately reduce new HIV infections, development of highly effective pre-exposure prophylaxis (PrEP) against HIV infection in women is necessary. Cellulose acetate phthalate (CAP) is a pH sensitive polymer with HIV-1 entry inhibitory properties. Dolutegravir (DTG) is an integrase strand transfer inhibitor with potent antiretroviral activity. DTG delivered in combination with CAP may significantly improve current PrEP against HIV. In the present study, the development of DTG-loaded CAP nanoparticles incorporated in thermosensitive (TMS) gel at vaginal pH 4.2 and seminal fluid pH 7.4 is presented as proof-of-concept for improved PrEP. Water–oil–in–water homogenization was used to fabricate DTG-loaded CAP nanoparticles (DTG–CAP–NPs). Size, polydispersity, and morphological analyses illustrate that DTG–CAP–NPs were smooth and spherical, ≤200 nm in size, and monodispersed with a polydispersity index PDI ≤ 0.2. The drug encapsulation (EE%) and release profile of DTG–CAP–NPs was determined by HPLC analysis. The EE% of DTG in DTG–CAP–NPs was evaluated to be ~70%. The thermal sensitivity of the TMS gel was optimized and the pH dependency was evaluated by rheological analysis. DTG release studies in TMS gel revealed that DTG–CAP–NPs were stable in TMS gel at pH 4.2 while DTG–CAP–NPs in TMS gel at pH 7.4 rapidly release DTG (≥80% release within 1 h). Cytotoxicity studies using vaginal cell lines revealed that DTG–CAP–NPs were relatively non-cytotoxic at concentration <1 µg/mL. Confocal microscopic studies illustrate that ≥98% cells retained DTG–CAP–NPs intracellularly over seven days. Antiretroviral drug loaded nanocellulose fabrications in TMS gel delivered intravaginally may enhance both microbicidal and antiretroviral drug efficacy and may present a novel option for female PrEP against HIV.

## 1. Introduction

Acquired immune deficiency syndrome (AIDS) caused by HIV infection is one of the insurmountable healthcare problems of the 21st century. Over 35 million people worldwide (1.3 million in the USA) are living with HIV-1, and, in 2015, 2.1 million new HIV infections were reported [1,2]. Of those newly infected individuals, 47% were women and 8% were children less than 15 years old [1]. Young people between the ages of 15 and 24 accounted for 35% of all new adult infections, with infection rates of young women in this age group accounting for 20% of the global sum of HIV infections [1,2]. Greater than 80% of HIV infections are contracted through sexual transmission and 86% of female transmission has been attributed to heterosexual intercourse [3,4]. HIV/AIDS remains the leading cause of death for pre-menopausal women worldwide [5]. Given international efforts to reduce the annual global HIV infection rates by 90% by 2030 [1], highly efficacious therapeutic and preventative HIV therapeutic options must be available to at-risk populations, particularly women.

Initially, natural or semi-synthetic macromolecular HIV-1 entry inhibitors were explored for PrEP of HIV infections. However, clinical trials revealed that macromolecular HIV-1 entry inhibitors alone such as cellulose sulfate, PRO-2000, and carrageenan sulfate do not offer significant protection from HIV infections [6]. Cellulose acetate phthalate (CAP) is an FDA approved low cost pharmaceutical excipient that is widely used for enteric coating of pharmaceutical formulations [7]. CAP is pH sensitive polymer that is stable at acidic pH and depolymerizes at pH higher than 6.2. Consequently, CAP is stable in the acidic vaginal environment [8]. CAP was found to interfere with the virility of HIV-1, HSV-1 and HSV-2 indicating its potential use as a topical microbicide [9]. CAP acts as a HIV-1 entry inhibitor (for R4 and R5 tropic viruses) by binding to gp 120 and inducing a conformational change in the trimeric gp120/gp41 complex leading to six-helix bundles that render HIV-1 unable to fuse with the host cell membrane [10,11]. CAP can also induce disintegration of HIV-1 by stripping envelope glycoproteins to cause reduced viral infectivity [10,11,12]. CAP was found to be active against HIV-1 in soluble and insoluble form suggesting that CAP can prevent HIV-1 infection in vaginal lumen (cervicovaginal mucus) as well as vaginal mucosa [12]. Vaginal application of a gel containing micronized CAP (13% *w/v*) did not alter vaginal pH, vaginal microflora, or integrity of vaginal epithelium and prevented SHIV infection in macaques [13,14,15]. However, in a phase 1 clinical trial, CAP infused gel was found to cause unacceptable vulvo-vaginal side effects due to very high osmolarity of gel [16]. Further in vitro studies showed that exposure to 0.05 mg/mL of CAP fibers resulted in neutralization of HIV-1. These CAP fibers were minimally toxic to vaginal epithelial cells at concentrations of up to 1.8 mg/mL [16].

Recently, antiretroviral drugs (ARVs) such as the reverse transcriptase inhibitors etravirine (ETR) and tenofovir disproxil fumarate (TDF) have been encapsulated into CAP electrospun fibers [17]. Complete neutralization of viral particles in a non-cellular environment was reported upon incorporation of 17.8% (wt TDF/wt CAP polymer) of TDF. However, a major challenge faced was the ability of and the time required to deliver drug to cells by CAP electrospun fibers [18]. Consequently, there is a need for an alternative formulation approach for combined PrEP delivered by CAP and ARVs.

We have already shown that CAP nanoparticles (NPs) can enhance anti-HIV efficacy when delivered in combination with antiretroviral drugs such as efavirenz [19]. Our synthesis of CAP nanoparticles delivering antiretroviral drug in an osmotically neutral thermosensitive (TMS) gel demonstrated in principle that nanocellulose-based fabrications can: (1) offer sustained release of drug; (2) improve cellular permeability and uptake; (3) improve chemical, enzymatic and metabolic stability of drug; and (4) improve local/systemic biodistribution of the drug [7,20]. We expand these preliminary studies to demonstrate here that nanoformulation of the pH sensitive, topical microbicide CAP in combination with antiretroviral drugs (ARVs) may prevent entry as well as integration of HIV-1 within the human genome, and may be a viable and potent option for HIV-1 prophylaxis [4,21].

Integrase is a key enzyme for integration of HIV-1 into host cell genome [22]. Since integrase has no human homolog, HIV-1 integrase strand transfer inhibitors (ISTIs) have become a primary treatment option. Dolutegravir (DTG) has been approved by the FDA for HIV-1 treatment [22]. DTG is a second-generation integrase inhibitor that has potent activity against wild-type HIV-1 (EC_50_: 0.51–1.6 nM and protein-adjusted EC_90_: 64 ng/mL) and can inhibit various strains of HIV-1 at nanomolar concentrations [23,24,25,26,27,28]. DTG solution is more effective than other ISTIs at inhibiting HIV-1 infection of peripheral blood monocytes (PBMCs) [23]. DTG presents a higher barrier against the development of resistance as compared to other ISTIs. DTG has a diminished rate of dissociation from the integrase enzyme in wild type HIV and HIV strains with the N155, Q148 or Y143 mutations [24,25,26,27,28]. Consequently, DTG retains activity against ISTI resistant HIV strains [24,25,26,27,28,29] and clinical trials have shown that DTG is capable of reducing viral load in patients harboring ISTI resistant HIV-1 strains [30,31]. DTG is also effective against nucleoside reverse transcriptase inhibitor (NRTI), non-nucleoside reverse transcriptase inhibitor (NNRTI) and protease inhibitor (PI)-resistant isolates [23]. All these features indicate that DTG could be a novel candidate for topical HIV PrEP delivery by CAP nanoparticles.

This is the first report of the synthesis and cellular tolerability of cellulose-based integrase strand transfer inhibitor nanofabrications designed for female PrEP. Additional studies on anti-HIV efficacy of these nanofabrications are underway.

## 2. Materials and Methods

### 2.1. Materials

From Sigma-Aldrich (St. Louis, MO, USA), CAP (*M*_W_: 2534.12), Poly(vinyl alcohol) (PVA) (*M*_W_: 13,000–23,000), Acetone, Acetonitrile (ACN), Potassium dihydrogen phosphate (KH2PO4), and Phosphate Buffered Saline (PBS) were purchased. Dimethyl Sulfoxide (DMSO) was purchased from Fisher BioReagents and Fisher Chemicals (Fair Lawn, NJ, USA). Fetal Bovine Serum (FBS), Trypsin and Penicillin-Streptomycin (Pen/strep) solution was purchased from Hyclone™ (Logan, UT, USA). Gibco™ Dulbecco’s Modified Eagle Medium (DMEM), Dolutegravir (DTG; 99% purity), Pluronic F127 (PF-127), and Rhodamine 6G dye (Rho6G; 99% purity) were purchased from FisherThermo Scientific (Rochester, NY, USA), Sequoia Research Products Ltd. (Pangbourne, UK), D-BASF (Edinburgh, UK), and Acros Organics (Geel, Belgium), respectively. Dolutegravir (DTG; 99% purity) was also obtained as a generous gift from ViiV Healthcare (Middlesex, UK). 3,3′-Dioctadecyl-5,5′-Di(4-Sulfophenyl)Oxacarbocyanine (DiO) dye, and DAPI dye were purchased from Life Technologies (Eugene, OR, USA). Paraformaldehyde (PFA) was purchased from Fisher BioReagents and Fisher Chemicals (Fair Lawn, NJ, USA). Keratinocyte-Serum Free medium (GIBCO-BRL 17005-042), human recombinant EGF, bovine pituitary extract, and calcium chloride were purchased from FisherThermo Scientific (Rochester, NY, USA). When appropriate, ultrapure water was used for all the experiments. All reagents were used as received without further purification.

### 2.2. Nanoparticles Preparation and Characterization

Drug or dye loaded CAP–NPs were prepared by interfacial polymer deposition method by water–in–oil–in–water (W–O–W) or oil–in–water (O–W) emulsion technique with some modifications [32,33,34,35]. Briefly, 50 mg of CAP was dissolved in the 5 mL Acetone (the organic phase) containing 50 mg of PF-127 as stabilizer. To formulate DTG loaded CAP–NPs (DTG–CAP–NPs), DTG (5 mg) in 2.5 mL of 0.5% PVA was added drop-wise on the above mentioned organic phase under constant magnetic stirring, followed by 5 min sonication having 10 s bursts (90% amplitude and 0.9 cycle), resulting in water–in–oil (W–O) emulsion. Further, the above W-O emulsion was subsequently added drop wise on to 10 mL 1% PVA solution under constant magnetic stirring, followed by 5 min sonication (at above mentioned setting) resulting in water–in–oil–in–water (W–O–W) emulsion. A similar method was followed to generate blank CAP–NPs (CAP–NPs) without using DTG. To formulate Rho6G loaded CAP–NPs (Rho6G–CAP–NPs), Rho6G (5 mg) was added to the above organic phase. The above organic phase was added drop-wise on to 10 mL of 1% PVA solution resulting in an oil–in–water (O–W) emulsion. The O–W emulsion was then sonicated (at above mentioned setting) for 5 min. For both formulations, the organic phase was eliminated by evaporation. Finally, the surfactants, free DTG or Rho6G, were washed off from NPs three times by dialysis using a dialysis-cassette (MWCO 20 kDa; Thermo Scientific; Rockford, IL, USA) with 2 L miliQ grade water (water with 18.2 mΩ resistance). NPs were then freeze-dried in the Millrock LD85 lyophilizer (Kingston, NY, USA).

For physico-morphological characterization, an appropriate amount of freeze-dried NPs were evaluated. For dynamic light scattering analysis (DLS), 5 mg/mL of respective NPs were dissociated in ultrapure water (with 18.2 MΩ resistivity at 25 °C). The NPs were then sonicated for 10 min. For size-distribution analysis, NPs were further diluted to 1 mg/mL and for surface charge distribution analysis NPs were further diluted to 2 mg/mL. The size, polydispersity index (PDI) and zeta potential of the NPs was characterized by using the ZetaPlus Zeta Potential Analyzer (Brookhaven Instruments Corporation, Holtsville, NY, USA). Five batches of NPs were analyzed to verify the reproducibility of the formulation method.

The percentage encapsulation efficiency (EE%) was evaluated by high performance liquid chromatography (HPLC) analysis (Shimadzu, Kyoto, Japan). However, for Rho6G EE% evaluation, UV–Visible absorbance analysis was performed by Nanodrop 2000c/2000 UV–Vis Spectrophotometers (Thermo scientific, Rockford, IL, USA) [32]. Briefly, 1 mg of DTG–CAP–NPs and Rho6G–CAP–NPs were disintegrated in 100 µL 40% DMSO and were spun (at 14,000× *g* for 5 min at 4 °C) filtered through Amicon^®^ Ultra Centrifugal filters (MWCO 30KDa; Merck KGaA, Darmstadt, Germany). DTG solution was used for the standard curve and a similar protocol was followed. Standards concentration ranges from 500 to 1.9 µg/mL were used to determine the standard curve (*r*^2^ = 0.99). HPLC analysis was carried out on Phenomenex C18 column (150 mm × 4.6 mm, 5 µm), using isocratic mobile phase (25 mM KH_2_PO_4_ 45%: ACN 55%) at a flow rate of 0.5 mL/min. UV absorbance was measured at 260 nm for both DTG and Rho6G. Retention times of DTG and Rho6G were 6.3 min and 8.4 min, respectively. The EE% by the following formula:

(1) $$\mathrm{EE}\%=\frac{\left(\mathrm{Amount}\;\mathrm{of}\;\mathrm{drug}\;\mathrm{entrapped}\;\mathrm{in}\;\mathrm{NPs}\right)}{\left(\mathrm{Amount}\;\mathrm{of}\;\mathrm{drug}\;\mathrm{added}\;\mathrm{to}\;\mathrm{the}\;\mathrm{emulsion}\right)}\times 100$$

To evaluate the morphology of the NPs, Scanning Electronic Microscope (SEM) imaging was performed as described previously [32,36,37]. Briefly, after filtering NPs suspension through a Whatman^®^ Nuclepore Track-Etch Membrane (~50 nm pore size), the air-dried NP-bearing membrane was mounted on the SEM stub and sputter coated with a thin layer (~3–5 nm thick) of chromium. The NPs on the membrane were imaged under a Hitachi S-4700 Field-emission SEM (New York, NY, USA).

### 2.3. Drug Release Study

To evaluate in vitro drug release efficiency, 5 mg of DTG–CAP–NPs were dissolved in 1 mL of 50 mM citrate buffer (pH 4.2) or in 10 mM PBS (pH 7.4), respectively. A 200 µL DTG–CAP–NP solution was collected at each respective time period (1 h, 1, 4 and 7 day). The solution obtained was spun (at 14,000× *g* for 5 min at 4 °C) to remove NPs and filtered through Amicon^®^ Ultra centrifugal filters (MWCO 30KDa; Merck KGaA, Darmstadt, Germany) for drug analysis. The DTG concentration was further evaluated by HPLC as described above. During data analyses, the volume correction factor was considered. The experiment was performed in triplicate for three independent experimental data sets. The released DTG concentration was evaluated by following equation:

(2) $${\%\;\mathrm{DTG}\;\mathrm{release}\;\mathrm{at}\;\mathrm{time}\;\prime t}_{n}\prime =\frac{\mathrm{Drug}\;\mathrm{amount}\;\mathrm{obtained}\;\mathrm{at}\;\mathrm{time}\;\prime t\prime }{\mathrm{Total}\;\mathrm{amount}\;\mathrm{in}\;\mathrm{NP}\;\mathrm{at}\;\mathrm{respective}\;\mathrm{volume}}\times 100$$

(3) $$\%\;\mathrm{actual}\;\mathrm{DTG}\;\mathrm{release}\;\mathrm{at}\;\prime t\prime =\left({\%\;\mathrm{DTG}\;\mathrm{release}\;\mathrm{at}\;\mathrm{time}\;\prime t}_{n}\prime \right)-\left({\%\;\mathrm{DTG}\;\mathrm{released}\;\mathrm{at}\;\mathrm{time}\;\prime t}_{n-1}\prime \right)$$

where “*t*” is the time interval (1 h, 1, 4 and 7 day) and “*t_n_*” represents corresponding time sequence number (i.e., 1 h, *t_n_* = 1; 1 day, *t_n_* = 2; etc.). 

### 2.4. In Vitro Uptake of CAP–Rhod6G/DTG–NPs Viewed by Confocal Imaging

VK2/E6E7 cells were dissociated from culture flasks and plated at 10^4^ cells per well on sterile four-chamber slides in supplemented VK2/E6E7 media. Slides were incubated overnight (O/N) at 37 °C and 5% CO_2_ to allow for adherence to the slide surface. CAP–Rhod6G–NP and Rhod6G solutions were diluted in 1 mL of sterile DI water to make a stock solution with a working concentration of 5 mg/mL. NPs were applied to cells at a final concentration of 1 μg/mL final concentration of DTG or Rhod6G in supplemented VK2/E6E7 media. After cells had been exposed to NPs, cells were fixed at 30 min and 7 days in 4% paraformaldehyde in PBS solution then washed in triplicate with 1× PBS three times. To stain the plasma membrane, DiO membrane stain (#V22886, Waltham, MA, USA) was applied at a dilution of 1:200 in Keratinocyte-Serum Free medium and incubation for 8 min at 37 °C. Plates were washed with 1× PBS three times. To stain the nucleus, cells were further incubated with DAPI (300 ng/mL) for 15 min, then washed twice with 1× PBS and mounted in Permafluor™ mounting media (#TA-006-FM, Thermofisher Scientific, Waltham, MA, USA). Cover-slipped slides were then sealed using nail polish and dried on a slide warmer. These slides were imaged in Creighton University’s Integrated Biomedical Imaging Facility on its IBIF Leica TCS SP8 MP Confocal Microscope at high magnification using a HC PL Apochromat 63 × 1.4 N.A. oil objective. To visualize the DAPI nuclear stain, DiO membrane stain, and the Rho6G CAP NPs, the excitation/emission spectra selected was 405/461 nm, 488/520 nm, and 530/552 nm, respectively. Confocal images were analyzed and orthogonal planar pictures were acquired from Leica LAS X Microscope Software (Wetzlar, Germany). 

### 2.5. Preparation of NP Dispersed in Thermosensitive (TMS) Gel

The TMS gel was prepared by following the method we described previously, with a few modifications [34]. Briefly, to prepare TMS gel of pH 4.2 and 7.4, a 30:0.7 ratio of Pluronic F127 to Pluronic F68 was dissolved in 50 mM Citrate buffer (pH 4.2) and 10 mM PBS (pH 7.4), respectively. The gelation was carried out at 4 °C. To prepare NP dispersed TMS gel, a respective amount of NPs were dissolved in respective pH buffer and thoroughly dispersed, followed by addition of TMS gel ingredients as mentioned above. Further, the above-mentioned gelation procedure was followed. All procedures were performed under aseptic condition.

### 2.6. TMS Gelation Property Analysis at Physiological Condition

To evaluate the viscoelastic properties of TMS gel, the thermogelation point and dynamic viscosity were examined. Dynamic rheological analyses were performed using an AR2000 rheometer (TA Instruments, New Castle, DE, USA). TMS gel measurements were performed using stainless steel cone/plate geometry (diameter: 40 mm; angle: 2°; gap: 50 µm). The torque ranged from 0.05 µNm to 200 µNm. To evaluate the thermogelation point of the TMS gel, the measurement was subjected to temperature ramping from 10 to 45 °C, under constant strain (0.1%) and oscillatory frequency (1 Hz). The results were evaluated as a function of temperature. The variation in elastic modulus *G*’ and viscous modulus *G*” were obtained from the phase angle from the Rheology Advantage data analysis software (provided along with the instrument).

To evaluate the real-time gelation property of the TMS gel at respective pH 4.2 or 7.4, the above rheological studies were performed in presence of stimulated vaginal fluid (VF) or seminal fluid (SF). To estimate reproducibility, all experiments were replicated at least three times independently. The stimulated VF or SF were prepared following previously published methods without modification [33]. Briefly, the 200 µL TMS gel at respective pH 4.2 or 7.4 was added to 200 µL VF or SF or VF plus SF, and were mixed well before dispensing on the rheometer slab at 10 °C (Section 3.2).

### 2.7. Vaginal Epithelial Cell Culture

Human vaginal epithelial cell lines (VK2/E6E7 ATCC^®^ CRL2616™) were obtained from American Type Culture Collection (Manassas, VA, USA) and cultured according to their instructions. Briefly, VK2/E6E7 cells were cultured to 75% confluency in Keratinocyte-Serum Free medium (GIBCO-BRL 17005-042) with 0.1 ng/mL human recombinant EGF, 0.05 mg/mL bovine pituitary extract, and additional calcium chloride 44.1 mg/L (final concentration 0.4 mM) (supplemented VK2/E6E7 media) at 37 °C and 5% CO_2_. Cells were sub-cultured every 3 days using 0.25% (*w/v*) Trypsin–0.03% (*w/v*) EDTA solution (#SV30031.01, Waltham, MA, USA).

### 2.8. Cytotoxicity Assay

To evaluate the DTG–CAP–NPs and DTG–CAP–NPs–Gel cytotoxicity to vaginal epithelial cells, the vaginal epithelial cell line, VK2/E6E7 cells, were used. For in vitro cytotoxicity experiments using VK2/E6E7 cells, cells (2 × 10^4^ cells/well) were placed into 96-well plates overnight in their respective media (described above). For each type of NPs and pH, where TMS gel is at pH 4.2 or pH 7.4, separate experiments were run. For each set, the following variables were tested: blank CAP–NPs, blank CAP–NPs in respective TMS gel pH 4.2 or 7.4, DTG solution, DTG solution in respective TMS gel pH 4.2 or 7.4, DTG–CAP–NPs, DTG–CAP–NPs in respective TMS gel pH 4.2 or 7.4, untreated (the negative cytotoxicity control sample) and 5% triton-X (the positive cytotoxicity control sample). Each treatment variable was added to the cells in triplicate wells and incubated at 37 °C, 5% CO_2_ over the course of 24, 48, or 96 h. The DTG concentrations in solution and entrapped in NPs was 10, 1, 0.1, 0.01, 0.001, 0.0001 μg/mL. At pre-specified intervals (24, 48, and 96 h), the cell viability was estimated by using the Cell Titer-Glo kit (Promega; Madison, WI, USA) following manufacturer’s protocol. Cell viability results were measured using a baseline subtraction method from the luminometer. The data presented were normalized to the untreated control (100% viability). The results were assessed using analysis of variance (ANOVA). Graphs present results from three independent experiments as means ± standard deviation (SD) or standard error (SE) as indicated.

### 2.9. Statistical Analysis

All experiments were performed in triplicate unless noted otherwise. Data analyses of nanoparticle physiochemical characteristics are presented as the mean ± standard deviation (SD). All other experiments are presented as the mean ± standard error (SE). Cellular data were analyzed by two-way analysis of variance (ANOVA) followed by post hoc (Tukey’s multiple comparison test) and Pearson’s correlation using GraphPad Prism 5 software (La Jolla, CA, USA). Significant differences were considered significant at *p* < 0.05 (*).

## 3. Results 

### 3.1. Cellulose Acetate Phthalate Nanoparticle (CAP–NP) Characterization

To examine the potential of CAP–NPs for sustained delivery of DTG, DTG–CAP–NPs were first fabricated and characterized for in vitro incorporation into TMS gel and for cellular viability assays. Rho6G–CAP–NPs were fabricated and characterized for cellular localization studies. The physiochemical characteristics of DTG or Rhod6G uploaded CAP–NPs at a neutral pH are summarized in Table 1. DTG–CAP–NPs and Rhod6G–CAP–NPs obtained by the modified O/W emulsion had a mean particle size of ~200 nm diameter and were monodispersed as indicated by a polydispersity index (PDI) < 0.2. Surface charge analysis showed that NPs were weakly negatively charged (<−30 mV). The encapsulation efficiency (EE%) of CAP–NPs for DTG loading was over 70%. The encapsulation efficiency (EE%) of CAP–NPs for Rhod6G loading was over 50%.

**Figure 1:**
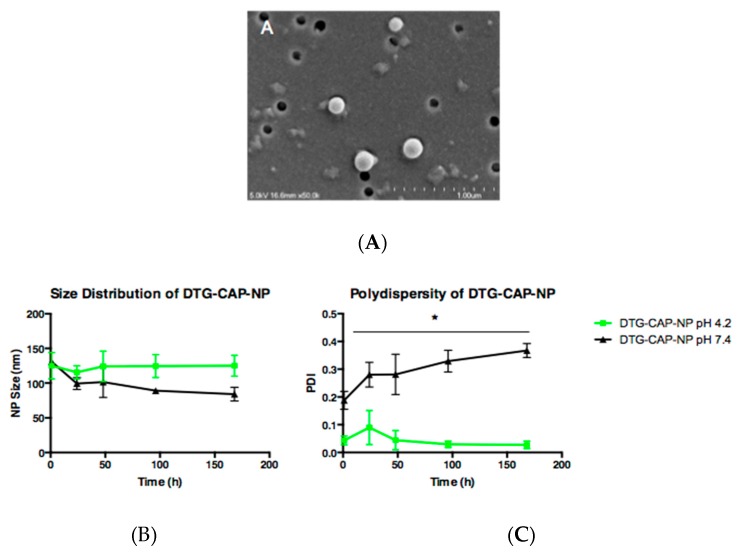
Characterization of DTG–CAP–NP. SEM of DTG-CAP-NP at pH 7.4. Scale bar shows 1.0 μm (**A**); Size distribution of CAP–DTG–NP for 168 h at pH 4.2 and pH 7.4 (**B**); Polydispersity index of CAP–DTG–NPs for 168 h at pH 4.2 and pH 7.4 (**C**); Error bars represent SD. * *p* < 0.05.

To determine the topography of DTG–CAP–NPs, scanning electron microscopy imaging was performed. Figure 1A shows an electron microscope image of DTG–CAP–NPs illustrating the uniform and smooth spherical surface. Since the vaginal environment is acidic ranging from pH 3.5 to pH 4.7 in >65% of post-puberty, pre-menopausal women and since CAP is responsive to pH, we compared the size and polydispersity of DTG–CAP–NPs in an average vaginal environment (pH 4.2) to a neutral pH that would be similar to seminal fluid (pH 7.4). To expand our preliminary data analyses evaluating NP size immediately upon sample preparation, we compared the size and polydispersity of DTG–CAP–NPs at pH 4.2 and pH 7.4 over a one-week (168 h) period (Figure 1B). Detailed analysis of the size of three additional batches of DTG–CAP–NPs demonstrates some variability and shows that overall the NPs are less than 200 nm in diameter and will function as nanoparticles. After one hour in a vagina-like pH 4.2 environment, DTG–CAP–NPs were 125 ± 19 nm. After one hour in a seminal-fluid-like environment of pH 7.4, DTG–CAP–NPs were 131 ± 8 nm. Over the course of one week the diameter of DTG–CAP–NPs at pH 4.2, did not significantly change. DTG–CAP–NPs were 125 ± 15 nm in size at 168 h (~0% change). Contrastingly, the diameter of DTG–CAP–NPs at pH 7.4 decreased ~35.9% from 131 ± 8 nm to 99 ± 15 nm within 24 h. After 24 h and up to 168 h, DTG–CAP–NPs decreased in size to 84 ± 17 nm. The polydispersity index (PDI) of DTG–CAP–NPs was also compared over the course of one week (Figure 1B) to estimate aggregation and disintegration properties of NPs at pH 4.2 or pH 7.4 over time. DTG–CAP–NPs in an average vagina-like pH of 4.2 maintained their monodispersity, as demonstrated by an averaged PDI of 0.04 ± 0.03 at one hour that was consistent with a PDI of 0.03 ± 0.02 at 168 h. The very low PDI values of DTG–CAP–NPs at pH 4.2 are likely due to the pH sensitivity of CAP. However, in the seminal fluid-like pH of 7.4, DTG–CAP–NPs exhibited an average PDI of 0.169 ± 0.06 at one hour that increased significantly to 0.367 ± 0.04 by 168 h (Figure 1B, *p* < 0.05). These data suggest that DTG–CAP–NPs maintain their integrity at an acidic pH of 4.2 and that CAP depolymerizes at physiological pH 7.4 reducing the size and increasing the diversity of NPs due to the formation of aggregates of disintegrated CAP polymer [38].

**Figure 2:**
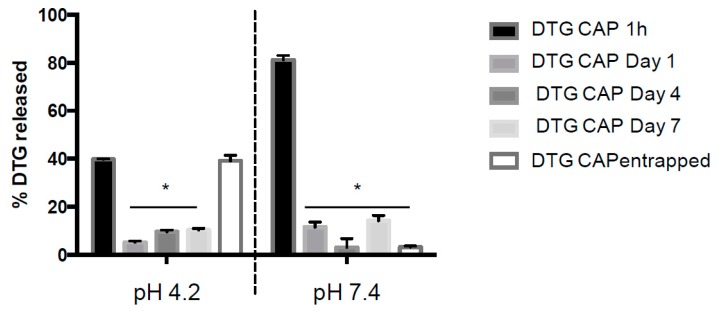
Percent of Entrapped DTG Released from DTG–CAP–NP. In vitro and HPLC analysis of DTG release from DTG–CAP–NPs over a one-week time at pH 4.2 and pH 7.4. DTG–CAP entrapped represents the amount of DTG remaining entrapped in the NP at the end of the one-week experiment. Error bars represent standard error of mean (SEM). * *p* < 0.05.

To evaluate the DTG release profile from DTG–CAP–NPs, the amount of DTG drug released over a one-week period from DTG–CAP–NPs at pH 4.2 and pH 7.4 was analyzed by HPLC (Figure 2). As expected, in physiological buffer (pH 7.4), DTG–CAP–NPs released 81.2% ± 0.02% of the entrapped DTG within one hour of incubation and, by the end of Day 7, only 3.2% ± 0.27% remained entrapped in the DTG–CAP–NPs. This finding supports our assumption that DTG-CAP-NPs disintegrate and rapidly deliver DTG at physiological pH similar to pH 7.4. Following one-hour incubation in pH 4.2 buffer, only 39.8% ± 0.10% DTG was released from DTG–CAP–NPs. Moreover, after one week at pH 4.2, 39.2% ± 1.33% of DTG was retained in DTG–CAP–NPs. The previously documented pH sensitivity of CAP [36] and our data showing a 36% decrease in size, increased PDI, and release of ~80% of DTG from DTG–CAP–NPs in one hour at pH 7.4, suggest that CAP disintegrated at pH 7.4. As a result, it is likely that DTG–CAP–NPs will release their content instantaneously when vaginal fluid of varying acidic pH intermixes with seminal fluid.

**Figure 3:**
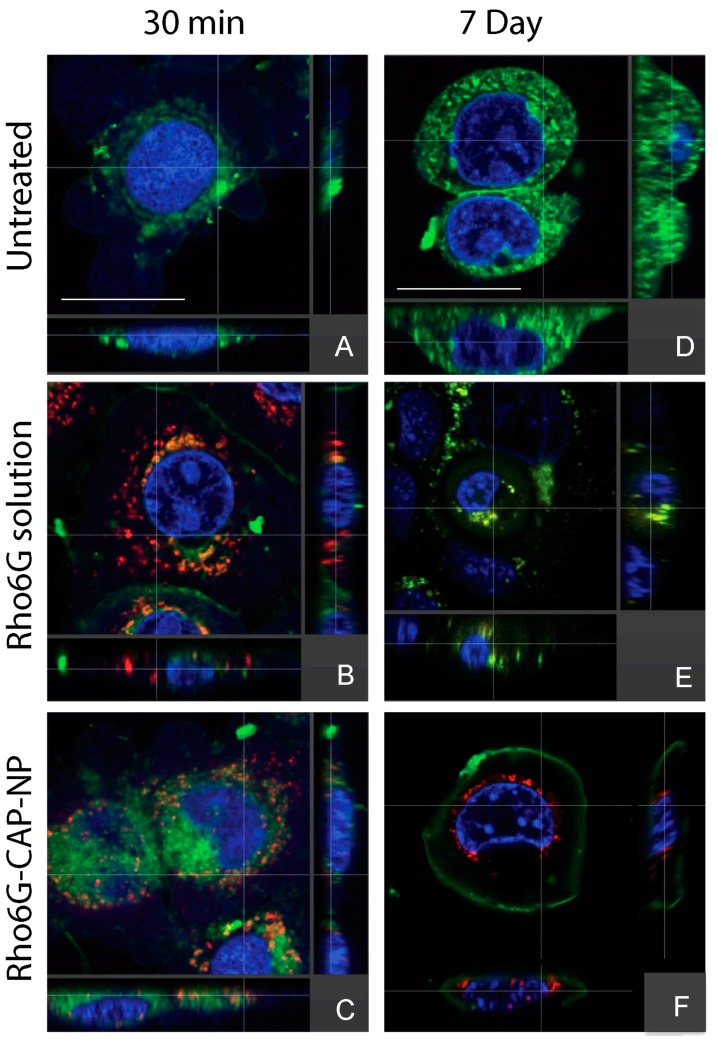
Translocation of Rhod6G Solution or Rhod6G–CAP–NPs to Vaginal Epithelial cells (VK2/E6E7). Rhod6G Solution (red) or Rhod6G–CAP–NPs at: 30 min (**A**–**C**); and seven days (**D**–**F**) post-treatment. Orthogonal Plane Images were obtained and evaluated by the IBIF Leica TCS SP8 MP Confocal Microscope at 63× with 5× computational magnification. DAPI: blue, DiO: green, Rhodamine: red. (**A**)/(**D**): Untreated, (**B**)/(**E**): Rhodamine Solution (1 μg/mL), (**C**)/(**F**): Rhod6G–CAP–NPs (1 μg/mL). Scale bar = 13 μm and is applicable to (**B**–**F**). For visualization of nanoparticles in print, scale bars are omitted from (**B**–**F**), and contrast and brightness were enhanced using imaging software.

**Figure 4:**
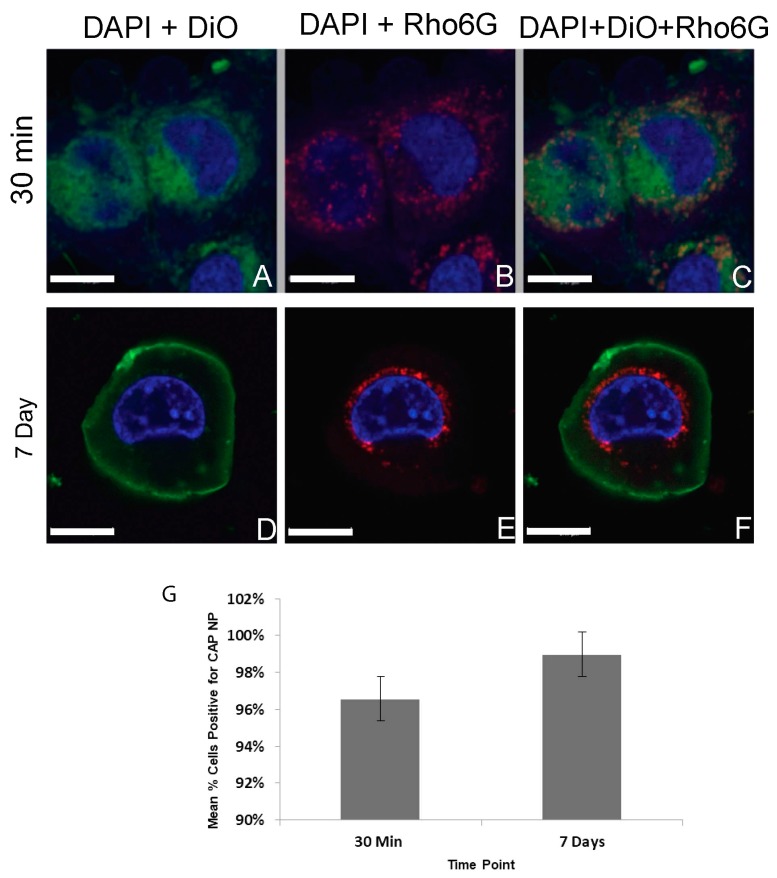
VK2/E6E7 vaginal epithelial cells treated with Rhod6G–CAP–NPs at (1 μg/mL) at: 30 min (**A**–**C**) and seven days (**D**–**F**) post-treatment. DAPI: Blue; DiO: Green; Rhodamine: Red. (**A**) DAPI + DiO; (**B**) DAPI + Rho; and (**C**) DAPI + DiO + Rho. (**G**) Mean percent VK2/E6E7 cells containing Rhod6G–CAP–NPs over time. 10× and 20× imaged on IBIF Leica TCS SP8 MP Confocal Microscope images analyzed in ImageJ (*n* = 4). For visualization of nanoparticles in print, contrast and brightness were enhanced using imaging software. (**G**) The percentage cells with Rho6G–CAP–NPs over seven days. Error bars reflect standard error of the mean, and the mean percent of cells containing Rhod6G–CAP–NP at 30 min and seven days was not significantly different (*p* > 0.05).

To investigate whether nano-encapsulation of DTG in CAP–NPs ensure cellular uptake and retention, we conducted cellular uptake studies and evaluated uptake efficiency by confocal imaging. To visualize under confocal fluorescent microscope, we used Rhod6G loaded NPs (Rho6G–CAP–NPs) and compared NP uptake with Rhod6G solution (Rhod6G Sol) at respective time points over one week. In Figure 3, Rho6G solution (free Rho6G dye) and Rhod6G–CAP–NPs are visualized as red fluorescence, the nuclei are stained with DAPI and are visualized as blue fluorescence and cellular membranes stained with DiO are visualized as green fluorescence. Both Rho6G solution and Rhod6G–CAP–NP treated cells show Rho6G fluorescence within 30 min of treatment. In both Rhod6G Sol and CAP–Rhod6G–NP treatments, co-localization (orange) of the Rho6G red fluorescence and the membrane green fluorescence suggests that the dye and/or NPs are proximal to or potentially incorporated into cellular membranes. Orthogonal view of the cell for both Rhod6G Sol and Rhod6G–CAP–NP suggest that delivery of solution and NP was intracellular (Figure 3). However, at Day 7 incubation Rho6G fluorescence was observed in Rhod6G–CAP–NPs treated VK2/E6E7 cells while in Rhod6G solution treated cells Rho6G fluorescence was not present. The orthogonal view of treated cells demonstrated that Rhod6G–CAP–NP show intracellular fluorescence near the nucleus at the seven-day time point (Figure 3). To further evaluate the above finding in cellular population, we next determined the percent of cells treated with these NPs that showed presence of Rho6G at 30 min and Day 7 of treatment (Figure 4). The number of Rhod6G–CAP–NPs positive cells as determined by red fluorescence at 30 min and seven day time points were divided by the total number of cells counted per field. Results show Rhod6G–CAP–NPs entered 96% ± 1.5% of cells at 30 min and were retained even after seven days at 98% ± 1.0% (Figure 4).

### 3.2. TMS Gelation Property Evaluation

One of the topical pre-exposure prophylactics (PrEP) for HIV infection is drug application vaginally or rectally that protect at-risk individuals from HIV and other sexually transmitted infections. Our strategy to fabricate DTG–CAP–NPs incorporated into TMS gel for use as a vaginal microbicide combines microbicide and ISTI to potentially provide enhanced PrEP for females. In our previous study, we found that CAP–NPs can be successfully incorporated into TMS gel to deliver ARV into cervical cells [19]. In the present study, with some modification, TMS gel was formulated at vaginal pH 4.2 and seminal pH 7.4. To evaluate the gelation properties of TMS gel samples at resting condition without disrupting the microstructure formation during gelation process, the viscoelastic measurements of TMS polymer gels near the sol-gel transition state were determined by dynamic rheological studies [33]. The thermogelation study revealed that TMS gel at pH 7.4 and pH 4.2 thermogelates at around 21.8 ± 1 °C and 15.2 ± 1.4 °C, respectively (Table 2). However, the presence of physiological fluids at the site of infection (i.e., VF and SF) does interfere with the thermogelation property of TMS gel. In the case of TMS gel at pH 4.2, the presence of VF or SF or both shows low to no gelation at 37 °C (Table 2). Moreover, even though the thermogelation temperature of TMS gel at pH 7.4 in presence of VF or SF or both is <37 °C, a clear shift of <10 °C of thermogelation temperature was observed (Table 2).

### 3.3. Cytotoxicity of CAP–DTG–NP

**Figure 5:**
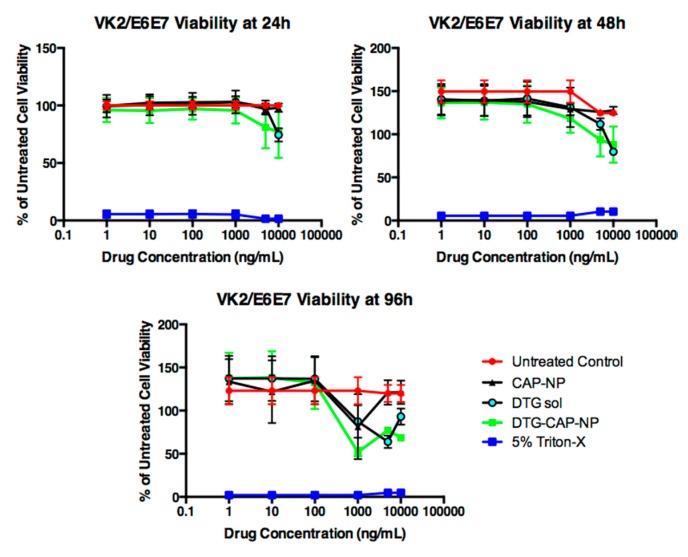
Vaginal epithelial VK2/E6E7 cell viability following treatment with DTG sol, CAP–NPs, and DTG–CAP–NPs at pH 7.4 (*n* = 6). Untreated control cells were used to indicate normal cell growth for three days in culture. Five-percent Triton-X treatment was used as a cytotoxicity control. Error bars represent SD.

DTG–CAP–NPs are a novel formulation designed to improve PrEP for HIV. As DTG–CAP–NPs are aimed to be delivered at the site of infection, i.e., on the vaginal epithelial layer, a primary question is whether DTG–CAP–NP fabrication can be delivered to vaginal cells without causing cytotoxicity. Cytotoxicity studies at pH 7.4 suggest that there is no significant difference in cellular viability when VK2/E6E7 are treated with CAP–NP, DTG–CAP–NP or DTG solution, when DTG drug concentrations are <1000 ng/mL. However, at concentrations ≥ 1000 ng/mL, moderate cytotoxicity was observed in DTG–CAP–NP and DTG solution treatments. At highest treatment concentration, i.e., 10,000 ng/mL, the viability at 24 h post-treatment was decreased by 23.01% ± 4.3% and 25.6% ± 4.3% for DTG–CAP–NP and DTG solution, respectively (Figure 5). Therefore, DTG–CAP–NPs were less cytotoxic to cells at 24 h than DTG solution by over 2.5% but this was not significant (*p* > 0.05). The 48 h post-treatment viability study illustrated that DTG–CAP–NP were less cytotoxic than DTG solution at 10,000 ng/mL and reduced viability by 36.52% ± 6.568% instead of 44.85% ± 6.568% as compared to control untreated cell viability. However, the improved viability of the DTG-CAP-NP treatment as compared to DTG solution of ~8% was not significant (*p* > 0.05) and both conditions are significantly more cytotoxic than untreated control cells. At 96 h, DTG–CAP–NP and DTG solution decreased cell viability when the DTG concentration was at 10,000 ng/mL by ~42.7% and ~56.0% as compared to control. Again, DTG–CAP–NPs at the highest concentration tested are less cytotoxic than DTG solution by ~13% but this difference was not significant using our analyses (*p* > 0.05). Treatment with 5% Triton-X induced cell death and is used as a negative control. Significant differences were determined using multiple comparison of two-way ANOVA followed by Tukey’s post-test.

### 3.4. Cytotoxicity of CAP–NPs in TMS Gel

**Figure 6:**
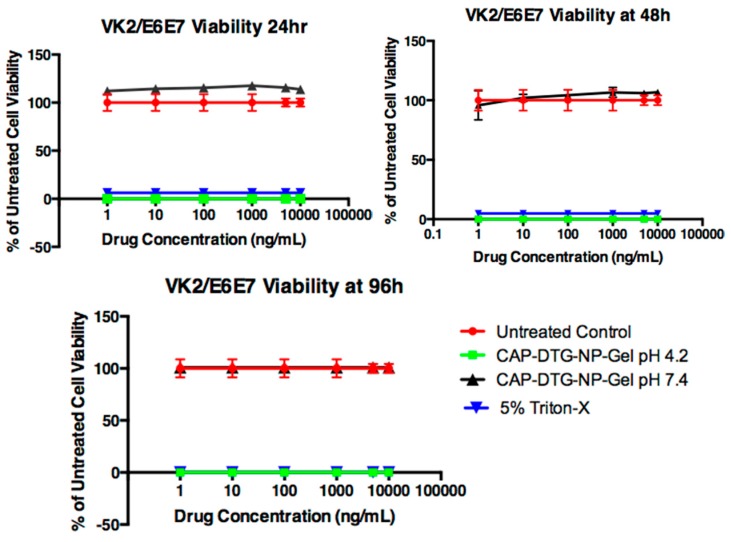
Vaginal epithelial VK2/E6E7 cell viability following treatment with DTG–CAP–NP–Gel at pH 7.4 (*n* = 6). Error bars represent SD.

To determine the cytotoxicity of DTG–CAP–NPs in pH 4.2 and pH 7.4 in gel, in vitro cytotoxicity assays were performed (Figure 6). Interestingly gel delivery of CAP–DTG–NPs (CAP–DTG–NP–Gel) at pH 7.4 is not cytotoxic to VK2/E6E7 cells even at the highest concentrations of DTG (*n* = 3, *p* > 0.05). Cell viability of CAP–DTG–NP–Gel at pH 7.4 was not significantly different from the viability of untreated control cells (*p* > 0.05). CAP–DTG–NP–Gel at pH 4.2 was cytotoxic to VK2/E6E7 cells and the acidic pH reduced viability similar to 5% Triton-X treatment. Both CAP–DTG–NP–Gel at pH 4.2 and 5% Triton-X treatments were significantly cytotoxic as compared to CAP–DTG–NP–Gel at pH 7.4 and untreated conditions (*p* < 0.05, Figure 6). Significant differences were determined using multiple comparison of two-way ANOVA followed by Tukey’s post-test.

## 4. Discussion

Topical microbicides that provide PrEP for females could reduce HIV-1 transmission dramatically [35]. CAP is FDA approved and widely used as a pharmaceutical excipient that displays microbicidal properties by inhibiting HIV in both its soluble and insoluble form by directly binding gp120 and interfering with p41Gag [9,11]. Additionally, the phthalate function group of CAP is pH sensitive (p*K*a of ~5.5) and undergoes a solution-to-gel phase transition as pH reaches neutral [36]. The microbicidal function and pH responsiveness of CAP suggests that CAP can be modified to effectively delivery antiretroviral drug for improved female HIV pre-exposure prophylaxis (PrEP). CAP, as well as several other topical microbicides that exhibited anti-HIV activity in preclinical trials, failed in clinical trials because the formulation caused irritation to female vaginal tissue and/or demonstrated a lack of efficacy [39,40,41]. Nanofabrication for delivery of CAP microbicide and drug may reduce the current limitations of topical microbicides and highly active antiretroviral therapy (HAART) that are challenged by tissue irritation, dosing complexities, and potential development of HIV resistance. We and others have developed CAP into nanofabrications for ARV delivery and improved PrEP [17,19] CAP electrospun nanofibers were designed to dissolve and release ETR or TDF within seconds to minutes after exposure to semen at pH 7.4. CAP fibers were minimally toxic to vaginal epithelial cells and could inhibit HIV virus in solution [17]. While fibers may offer the advantage of leak-free delivery system, fibers are challenged by their capacity to rapidly and effectively deliver drug to cells. Nanoparticles (NP) offer sustained release of drug and improved stability, permeability, cellular uptake, and local/systemic biodistribution of drug [7,20]. NPs that are readily taken up into cells and sustain drug delivery, particularly sustained delivery of drugs working prior to viral integration, may offer significantly improved PrEP.

The HIV-1 integrase strand transfer inhibitor (ISTI), DTG is a second-generation integrase inhibitor that has potent activity against wild-type HIV (EC_50_: 0.51–1.6 nM) and can inhibit various strains of HIV at nanomolar concentration [23,24,25,26,27,28]. DTG presents a high barrier against development of resistance as compared to other ISTIs, e.g., raltegravir (RAL) or elvitegravir (EVG). DTG retains activity against RAL and/or EVG resistant HIV strains [24,25,26,27,28,29] and clinical trials have shown that DTG is capable of reducing viral load in patients harboring RAL and/or EVG resistant HIV-1 strains [30,31]. DTG is also effective against nucleoside reverse transcriptase inhibitor (NRTI), non-nucleoside reverse transcriptase inhibitor (NNRTI) and protease inhibitor (PI)-resistant isolates [23]. We hypothesized that DTG is an excellent candidate for topical HIV PrEP delivered by CAP–NPs for improved female PrEP.

For the first time, we present the synthesis of CAP–NPs encapsulating the ISTI DTG. DTG–CAP–NPs were synthesized using O/W method to form a monodispersed (PDI < 0.2) population of NP averaging ~200 nm diameter. DTG–CAP–NPs were weakly negatively charged (<−30 mV). The encapsulation efficiency (EE%) of CAP–NPs for DTG loading was over 70%. The size, polydispersity index and EE% should allow for effective delivery of the ISTI DTG to cells over time (Table 1). DTG–CAP–NPs have a uniformly, smooth surface and demonstrate expected pH sensitivity over time (Figure 1A,B). While the vaginal pH among women varies depending upon age and ethnicity, DTG–CAP–NP synthesis was optimized for the average vaginal pH of 4.2 (range approximately pH 3.8–4.5) and pH of seminal fluid at pH 7.4 as proof-of-concept in these in vitro studies. The average pH for the vaginal environment in the majority (~65%) of post-puberty, pre-menopausal women was estimated to be approximately pH 4.2. At vaginal pH 4.2, the DTG–CAP–NPs do not show any significant change in their average size or PDI values. However, at physiological pH similar to that of seminal fluid (pH 7.4), DTG–CAP–NPs significantly decrease in size and polydispersity increases, demonstrating the depolymerization of the CAP polymer leading to release of DTG. The capacity to release DTG is evident from the DTG release studies (Figure 2). At pH 7.4, DTG–CAP–NPs released over 80% of the entrapped DTG and only ~3% remained entrapped in DTG-CAP-NP after one week. However, at pH 4.2, DTG–CAP–NPs released DTG < 40% in 1 h which could be attributed to increase in surface tension on NPs upon resuspension after freeze drying [42]. DTG–CAP–NPs shows retention of 40% of DTG even after one week. These data suggest that DTG from DTG–CAP–NP could be instantaneously released onto vaginal epithelial cells when the vaginal pH becomes neutralized by the seminal fluid. Delivery of DTG to cells is necessary for the ISTI to function against HIV. We demonstrated using confocal imaging that Rhod6G–CAP–NP were readily taken up into >95% of vaginal epithelial cells at 30 min and throughout the seven-day experiment (Table 1, and Figure 3 and Figure 4). Rhod6G–CAP–NPs were seen in the cytoplasm and near the nucleus of cells. While Rhod6G solution alone can be observed in cells at 30 min, Rhod6G solution is not observed in cells over a seven-day experiment. These data suggest that CAP–NPs can offer sustained delivery of DTG to cells over time and future studies are underway to confirm the delivery of DTG intracellularly. Further, since a body of work suggests that mucosal epithelial cells may provide a reservoir for HIV, delivery of microbicide and ISTI to vaginal epithelial cells directly could increase PrEP function [35].

The aim of present study was to develop a local NP delivery system that can potentially prolong retention of DTG–CAP–NPs at the target site. To reach this aim, a novel TMS gel fabrication was optimized at both pH 4.2 and 7.4, to maintain DTG–CAP–NP colloidal stability upon incorporation into TMS gel and to target specific drug release during the a putative time of infection. TMS gel composition was optimized to ascertain that TMS gel thermogelates at around 37 °C (Table 2). TMS gel fabrication provides a mechanism for vaginal application and delivery of pH sensitive, DTG–CAP–NPs to vaginal cells. Thermogelation upon application of DTG–CAP–NPs in TMS gel (DTG–CAP–NP–Gel) should allow maintenance of NPs at the vaginal epithelium over time. In vivo imaging studies determining the sustained delivery of NPs in gel to cells are underway. Since vaginal epithelial cells would be in direct contact with DTG–CAP–NPs via gel delivery, we examined the cytotoxicity of DTG–CAP–NPs and DTG–CAP–NP–Gel to vaginal epithelial cells (Figure 6). DTG–CAP–NPs in solution and in gel were not cytotoxic to vaginal epithelial cells at concentrations of DTG < 1000 ng/mL. Some cytotoxicity was seen when DTG–CAP–NPs were delivered in solution to vaginal epithelial cells and the DTG concentration is >1000 ng/mL (Figure 6). Our viability assays demonstrate that DTG–CAP–NPs at the highest treatment concentration of 10,000 ng/mL trend toward being increasingly less cytotoxic than DTG solution. The CAP–NP fabrication method is likely to provide a mechanism for drug delivery that is more tolerated by cells over time. DTG–CAP–NP–Gel at pH 4.2 were cytotoxic to vaginal epithelial cells since these modified cells are pH sensitive and must be cultured at physiological pH. Cell death at 96 h in the TMS gel at pH 4.2 is due to the response of these cells to acidic pH and the absence of the normal tissue environment. However, studies using vaginal tissue explants and animals are underway to determine the potential for tissue irritation following vaginal application of DTG–CAP–NP–Gel at pH 4.2. Importantly, no cytotoxicity was seen when DTG–CAP–NP–Gel were delivered to vaginal epithelial cells at pH 7.4 as compared to untreated control conditions (Figure 6). Previous studies using monkey model systems showed that vaginal application of a gel containing micronized CAP (13% *w/v*) did not alter vaginal pH, vaginal microflora, or integrity of vaginal epithelium and prevented SHIV infection [13,14,15] strongly suggesting CAPs usefulness in female PrEP. However, it is important to note that CAP gel fabrications for human delivery have required further study due to tissue irritation in women [16]. While this irritation was determined to be due to osmolarity of the gel use of DTG–CAP–NP–Gel for female PrEP would require investigation of potential affects on vaginal pH, micoflora, tissue irritation, and efficacy.

The known pH sensitivity of CAP [38] and our data at pH 7.4 suggest that pH values more basic than 5.5 will lead to depolymerization of DTG–CAP–NPs. In the presence of seminal fluid, the human female vaginal environment may be neutralized along a spectrum of pH less basic than the normal vaginal environment and more acidic than pH 7.4. While examining the exact extent of depolymerization of every potential basic pH is beyond the scope of this initial study, these studies strongly suggest that DTG–CAP–NPs are likely to disintegrate in neutral pH where seminal fluid and the risk of HIV virion delivery is most likely. Further, DTG–CAP–NPs are likely to generate a sudden burst and release of DTG to provide effective protection from HIV infection. TMS gel fabrication delivers CAP’s microbicidal properties and DTG’s integrase inhibitor anti-HIV properties using NP design in a osmotically neutral temperature sensitive gel to potentially block both cell-free and cell-associated HIV at the vaginal epithelium. Future studies will be focused on determining the penetration properties and anti-HIV efficacy and safety of DTG–CAP–NP–Gel using both ex vivo and in vivo model systems.

## 5. Conclusions

CAP–NPs may provide an important modality for the delivery of both microbicide function and ARV drug for female PrEP. In this study, CAP–NPs were fabricated at nanoscale, loaded with the integrase strand transfer inhibitor DTG, incorporated into thermosensitive gel, and delivered to a vaginal epithelial cell line. In vitro studies suggest that DTG–CAP–NPs can efficiently deliver both the microbicide activity of CAP and ARV activity of DTG to cells when the vaginal environment undergoes a pH transition associated with exposure to seminal fluid. Further studies will focus on establishing ex vivo and in vivo proofs for improved female PrEP mediated by CAP nanofabrications.

## Figures and Tables

**Table 1:** Physiochemical Characterization of CAP Nanoparticles.

CAP–NP Sample	Size (nm)	Surface Charge (mV)	Polydispersity Index (PDI)	EE%
DTG–CAP–NP	212.62 ± 20.8	−25.046 ± 3.4	0.189 ± 0.025	71.72 ± 7.5
Rhod6G–CAP–NP	164.5 ± 40.6	−27.15 ± 0.39	0.166 ± 0.0049	53.9 ± 6.33

Data presented as mean ± standard error of mean of six different NP batches (*n* = 6).

**Table 2:** Thermogelation property evaluation of TMS gel.

Sample	Vaginal Fluid (VF)	Seminal Fluid (SF)	TMS Gel:VF or SF Ratio	Thermogelation Temp. (°C)
TMS gel (pH 4.2)	−	−	-	21.8 ± 1
TMS gel (pH 4.2) in VF	+	−	1:1	37.9 ± 4.2
TMS gel (pH 4.2) in SF	−	+	1:1	>40
TMS gel (pH 4.2) in VF + SF	+	+	1:1	38.6 ± 2.9
TMS gel (pH 7.4)	−	−	-	15.2 ± 1.4
TMS gel (pH 7.4) in VF	+	−	1:1	36.4 ± 0.23
TMS gel (pH 7.4) in SF	−	+	1:1	35.7 ± 2.6
TMS gel (pH 7.4) in VF + SF	+	+	1:1	29.2 ± 6.7
